# A Survey of Analytical Techniques for Noroviruses

**DOI:** 10.3390/foods9030318

**Published:** 2020-03-10

**Authors:** Lingling Liu, Matthew D. Moore

**Affiliations:** 1 Department of Agricultural and Biosystems Engineering, Iowa State University, Ames, IA 50011, USA; 2 Department of Food Science, University of Massachusetts, Amherst, MA 01003, USA

**Keywords:** human norovirus, detection, review

## Abstract

As the leading cause of acute gastroenteritis worldwide, human noroviruses (HuNoVs) have caused around 685 million cases of infection and nearly $60 billion in losses every year. Despite their highly contagious nature, an effective vaccine for HuNoVs has yet to become commercially available. Therefore, rapid detection and subtyping of noroviruses is crucial for preventing viral spread. Over the past half century, there has been monumental progress in the development of techniques for the detection and analysis of noroviruses. However, currently no rapid, portable assays are available to detect and subtype infectious HuNoVs. The purpose of this review is to survey and present different analytical techniques for the detection and characterization of noroviruses.

## 1. Introduction

Human noroviruses (HuNoVs) are the leading cause of foodborne illnesses in the United States and lead to around 21 million cases of acute gastroenteritis annually, resulting in more than 70,000 hospitalizations and nearly 800 deaths [1]. The economic impact from foodborne and waterborne outbreaks of NoV illnesses is estimated to be $5.8 billion annually in the U.S. [2]. Approximately 5% of people among all ages are infected by HuNoV every year, according to the surveillance data from Netherlands, UK and USA [3]. HuNoVs are transmitted through the fecal-oral route, aerosolized vomitus, contaminated water or food, fomites, and direct person-to-person contact [4]. They are very persistent in the environment, being resistant against freezing/thawing (at least 14 cycles), drying, low pH (gastric pH 3–4) and common chemical disinfectants [5,6]. HuNoVs have particularly been shown to be able to survive for long periods of time in various food products, environmental water, and on contact surfaces [7,8,9,10]. The infection course of HuNoVs is all year long, though it is more often reported during the winter and early spring months, possibly due to the tendency for people to congregate in enclosed environments and take less exercise [4].

Noroviruses (NoVs) are members of the Norovirus genus within the Caliciviridae family. With a size of around 27~38 nm and a genome length of around 7.4~7.7 kb, NoVs are non-enveloped viruses with a single-strand, positive-sense RNA genome inside a protein capsid shell. Among the six genogroups of NoV, genogroup I and II (designated GI and GII) are of the greatest interest as they are the most common genogroups that infect humans. To date, there are at least nine genotypes of HuNoVs in GI and 22 in GII, which constitutes over 150 strains [11,12]. HuNoVs have a wide degree of antigenic and genetic variation. Based on the differences of the amino acid sequences for the major capsid protein of noroviruses, the variations between genogroups, genotypes, and strains are 44.9–61.4%, 14.3–43.8% and 0–14.1%, respectively [13]. Among all the different forms of HuNoVs, GII.4 is the most prevalent genotype across the world, which accounts for around 80% of all norovirus outbreaks since 2002 [14]. GII.4 NoVs comprise the majority of norovirus illnesses, and undergo substantial antigenic variation via recombination and mutation, resulting in a new pandemic GII.4 strain circulating every 2–4 years [15].

The large antigenic variations of HuNoV among genotypes and genogroups are one of the primary reasons why NoV vaccines have still yet to be developed. Other factors that have complicated the design of a vaccine include the lack of appropriate modeling, an unknown duration of protection by the vaccines, few human challenge studies, and complex patterns of vaccine performance due to unknown pre-exposure history [15]. Since no vaccine is available, the only effective way to mitigate HuNoV outbreaks is through prevention, early detection, and control. Due to the highly contagious nature of HuNoVs, once an outbreak starts, it is very important to identify the virus and its source immediately in order to control the damage [1]. However, significant technical challenges exist for the development of rapid assays with high sensitivity and specificity, especially for infectious HuNoVs. The current gold-standard reverse transcription-polymerase chain reaction (RT-PCR) method lacks portability, takes ≥ 40 min, is sensitive to complex matrices, and is unable to differentiate infectious from non-infectious HuNoV. Hence, the development of a rapid or near real-time detection method for HuNoVs has become even more necessary. Although challenges still exist, much progress has been made in the area of detection and biochemical analysis of noroviruses since their discovery nearly half a century ago. This review will survey past and present norovirus detection and analytical techniques. In general, detection techniques for NoVs can be grouped into ligand-based, nucleic acid-based, biosensor-based, microarray-based, omics-based and others. Comparisons among different types of methods for HuNoV detection have been summarized in Table 1.

## 2. Ligand-Based Detection Techniques

### 2.1. Ligands

Ligands that can bind with HuNoVs are key components in many assays for HuNoV detection. One of the more pressing challenges presently is the lack of a ligand that is broadly reactive enough to bind with all HuNoV genotypes. However, some ligands, including histo-blood group antigens (HBGAs), porcine gastric mucins (PGM) (containing HBGAs), aptamers and monoclonal antibodies (mAbs), have been widely used for the detection of HuNoVs. Each of these ligands can bind to some specific genotypes or strains of HuNoVs but not all, and each has their own limitations. The comparison of these four ligands is shown in Table 2.

#### 2.1.1. Histo-Blood Group Antigens

Histo-blood group antigens (HBGAs) are suggested to be receptors or co-receptors for HuNoV infection. HBGAs which include ABO, secretor and Lewis antigens are highly polymorphic [23]. They are widely present in red blood cells, mucosal epithelial cells and free antigens in body fluids, such as blood, saliva, milk and the intestinal contents [24,25]. Type A-like HBGA is also found in oyster gastrointestinal cells and responsible for the binding with recombinant norovirus-like particles (NoV VLPs) [26]. One inherent advantage of utilizing HBGAs in detection is that less noninfectious viral particles will be detected. HBGAs are commonly used as a reagent in removing signal from noninfectious viral particles under the assumption that if a viral particle is unable to bind its cellular receptor/co-receptor, it is not likely to infect a host cell.

Binding between HuNoVs and HBGAs is strain-specific [27]. At least eight binding patterns have been reported, which can be divided into two major binding groups based on shared HBGA targets. These include the A/B binding group (which mainly recognizes the A/B/H epitopes) and the Lewis binding group (which only recognizes the Lewis epitopes) [28,29]. HBGA-binding interfaces include a central binding pocket (CBP) and a variable surrounding region. The CBP is highly conserved and it interacts with a common major binding saccharide of HBGAs, while the surrounding region is very flexible, which allows for the binding of HuNoVs to diverse saccharides of HBGAs [23]. The binding between HBGAs and HuNoVs involves multiple epitopes, and is essentially a protein–carbohydrate interaction, with the amino acids in the protruding domain of the viral capsid interacting with oligosaccharides on HBGAs [30]. However, the mechanism is very complex, since several factors contribute to the binding interactions, including capsid loop movements, HBGA alternative conformations, and rotations. In particular, the capsid loop can be repositioned to allow for HBGA binding, which involves hydrogen bonds and water-mediated bonds [31].

HBGAs as receptors for HuNoVs can also be further described by the fact that HBGA-expressing bacteria can aid in the cultivation of HuNoVs in a B cell line [32]. In addition, different NoV strains have variable binding abilities to HBGA-expressing bacteria [32,33,34]. Even though almost all HuNoVs bind to HBGAs, some strains of GI VLPs, GII.1 VLPs and GII.14 VLP do not bind to any type of HBGAs nor any saliva [28,35]. For these strains, it is suggested that some other receptors may be involved in the binding process [36]. In addition, HBGAs have the shortcoming of low specificity as HuNoV receptors, since many other viruses (including rotavirus and rabbit haemorrhagic disease virus) and bacteria (e.g., *Escherichia coli*) also recognize HBGA as receptors for attachment [20]. As an example, spike protein VP8* from major human rotavirus genotypes (P [4], P [6] and P [8]) has been reported to bind with H type 1 HBGA [37].

Since HBGAs are terminal carbohydrate structures present on the sugar chains of oligosaccharides, glycans (containing HBGA), glycoconjugates (including glycolipids and glycoproteins) and saliva, have also been incorporated in the study of HBGAs with NoVs [38]. It has been reported that NoV VLPs bind to glycosphingolipids (GSL) in a strain-specific manner where Norwalk virus (NV) VLPs bind to A, H, and difucosylated Lewis, but not B histo-blood group active GSL [39,40]. Similarly, human milk glycans (human milk oligosaccharide (HMOS)) and HMOS-based neoglycoconjugates (including neoglycoproteins and oligosaccharide-glycine derivatives) can bind with NoV VLPs (VA387 and Norwalk) in a strain-specific manner and inhibit VLPs binding to their host receptors [41]. A study conducted by Rydell et al. [42] showed that aside from secretor-gene dependent α1,2-fucosylated carbohydrates, some HuNoV GII strains can also recognize sialyl Lewis x neoglycoproteins as binding receptors.

#### 2.1.2. Porcine Gastric Mucin

Porcine gastric mucin (PGM) has been widely used as a model for human gastric mucin in studies, since it shows similarities in terms of anatomy, physiology and sequence [43,44]. Both human and porcine gastric mucin have been reported to have a “necklace”-like structure under atomic force microscopy (AFM) [45]. PGM is composed of protein (20%) and carbohydrates (such as hexosamine (37%), hexoses (27%), fucose (10%) and sialic acid (6%)) [21]. The presence of HBGA (type A, H type 1 and Lewis b) in PGM (type III, Sigma-Aldrich) has been determined by enzyme-linked immunosorbent assay (ELISA) with the incorporation of anti-HBGA antibodies [46]. Due to the fact that PGM can be more readily obtained and can be less costly than purified HBGAs, numerous reports exist utilizing PGM for capture of noroviruses. Like HBGAs, PGM has the potential added benefit of selecting less nonviable viral particles. Specifically, this ability is premised upon the assumption that if a viral particle is not capable of binding a putative co-factor/receptor necessary for infection, then it will not be able to infect a cell. Further, PGM has the added benefit of containing multiple HBGA types and sialic acid, making it capable of binding and capturing a broader range of noroviruses. This is because specific HBGA binding profiles differ among different norovirus genotypes and strains [31,47]. The binding of NoV VLPs with HBGA on epithelial cells of porcine gastrointestinal tissue has been observed by using fluorescent labelled anti-HBGA antibodies and secondary antibodies through confocal microscopy [48].

PGM binds to NoV VLPs, as well as inhibits the binding of NoV VLPs to HBGAs and Caco-2 cells [49]. Specifically, NoV VLPs could be efficiently captured by PGM coated on plates. However, the binding of NoV VLPs to PGM can be inhibited by HBGA in saliva, Lewis b and Lewis d synthetic oligosaccharides. In a study conducted by Tian et al. [50], all GI (8 strains) and 85% of GII (11 strains) recombinant NoVs tested were successfully captured and concentrated via PGM conjugated magnetic beads (PGM-MB). However, as not all NoVs bind to HBGAs, not all can be captured by PGM as well. For example, some NoV VLPs of GII.4 strains (Sakai, Hunter and Bristol) could not be concentrated by the PGM-MB assay [50]. Although PGM is broadly available and reactive among HuNoV genotypes [26], it has low specificity since it can also bind to other viruses and bacteria [22].

#### 2.1.3. Antibody

Antibodies are often recognized as the most popular bio-recognition elements due to their sensitivity and specificity [51]. Antibodies (including polyclonal, monoclonal and recombinant antibodies) as well as their fragments (antigen-binding fragment and variable domain) are often used in immunological diagnostics and biomarker detection [52]. Polyclonal antibodies (pAbs) of NoVs were found to be highly specific for the immunized genotypes, which hindered the development of immunological diagnosis. Thus, interest was turned towards the development of mAbs, which are also more stable than pAbs in terms of their application in rapid immunological assays [53]. Although numerous commercially available antibodies exist (12 available from Abcam against GI and GII; with others available from ThermoFisher #MA1-7405, and Sigma-Aldrich #MABF2097, among others), they will not be discussed for the purposes of this review.

Monoclonal antibodies against HuNoV can be produced from BALB/c mice (hybridoma cell line) with HuNoV VLPs [54]. Broadly reactive mAbs are often preferred for HuNoV detection from clinical samples. Previously reported broadly reactive mAbs can be classified into several groups based on their epitope properties [54]. The first group of mAbs are cross-reactive, since they recognize the inter-genogroup cross-reactive linear epitopes on the shell or protruding domain. The second group of mAbs are genogroup-specific, capable of recognizing the intra-genogroup cross-reactive conformational epitopes, and the third group are strain-specific [54].

mAb NV23, which recognizes an epitope on the VP1 protruding domain (residues 453~472) of GI, GII, and GIV NoVs, was shown to be cross-reactive by both surface plasmon resonance (SPR) and ELISA methods [55]. Specifically, it was able to detect HuNoV VLPs from 16 genotypes by sandwich ELISA, and detect HuNoV from stool specimens with a Ct value <31, as shown by Kou et al. [16] Studies incorporating two different antibodies have shown to be able to detect more HuNoV genotypes. In particular, another study conducted by Kou et al. [56] combining mAb NV23 and single-chain variable fragments (scFv) (HJT-R3-A9 antibody) for NoV capture and detection by a sandwich ELISA assay showed that all 25 NoV genotypes from stool samples could be identified with a Ct value of <31 and a limit of detection (LOD) of 1 ~ 10 ng/well for 8 GI and 13 GII genotypes, except 50 ng/well for GII.7 strains. In addition, Hurwitz et al. [57] identified two scFvs (NJT-R3-A2 and NJT-R3-A3) that could detect NoV from infected clinical stool samples [57]. The LOD of NJT-R3-A2 scFv for the detection of GI.1 and GI.7 VLPs were 0.1 and 0.2 ng, respectively. Zheng et al. [58] also identified two mAbs (8D8 and 10B11) that could bind to all eight major VP1 capsid proteins of NoV with varied binding affinities. However, mAb 8D8 did not bind with intact VLPs in solution.

Nanobodies, known as the smallest fragments of antibodies with a single-domain [59], have raised researchers’ interest and have been used for NoV detection. The advantages of nanobodies over antibodies include the fact that nanobodies are more stable than conventional antibodies, and the former can be produced in large quantities with the atomic resolution structure of binding pockets easier determined than the latter [60]. The nanobody can bind to the top, bottom and side of NoV protruding domain [61]. In a study conducted by Koromyslova and Hansman [62], a nanobody (nano-85) was broadly reactive, as it could bind to GII. 4, GII. 10, and GII. 12 NoV, as well as being able to detect NoV from stool samples via ELISA. Another study conducted by Doerflinger et al. [60], showed that nano-85 was able to bind to four GII NoV VLPs (including GII.4, GII.10, GII.12 and GII. 17), and the nanobody-based lateral flow immunoassay was able to detect GII.4 outbreak specimens, with a sensitivity of 80% and specificity of 86% in ~5 min. Interestingly, NoV VLPs appeared to fall apart if incubated with nanobodies, since nanobodies bind so close to the icosahedral contacts of the virus. Specifically, nanobodies can cause NoV capsid morphological changes, resulting in the degradation of capsid protein and exposure of viral RNA [61].

Synbodies which are synthetic bivalent ligands with two 15- to 20-mer peptides have similar affinities and specificities to antibodies [63]. A series of synbodies has been developed by using NoV GII.4 VLPs with dissociation constants (Kd) < 10 nM [64]. The synbodies were reported to be broadly cross-reactive with NoV VLPs from multiple GI and GII genotypes, but they are not reactive to all. By using a synbody (ASU1052)-based magnetic bead capture assay, NoV could be concentrated from dilute stool solutions [63]. The utilization of a synbody (ASU1052)-based magnetic bead capture assay could result in 1000-fold increase in the sensitivity of a low-cost eye-readable assay for the detection of NoV (GII.4 Sydney), from clinical stool samples with a LOD of 270 zM in 3 h [63].

Though antibodies have been widely used in the detection of HuNoVs in a number of formats, including immunoassays and biosensor-based assays, currently developed antibodies are only partially broadly reactive towards HuNoVs. Specifically, 29 genotypes of HuNoVs exhibit 142 dissimilar antigenic behaviors. In addition, certain strains of HuNoVs undergo antigenic drift over time [11].

#### 2.1.4. Aptamers

Aptamers are small single stranded DNA or RNA oligonucleotides (usually 20 ~ 60 nucleotides) that can fold into well-defined three-dimensional (3D) structures and bind to target molecules with high affinity and selectivity [19,65]. Through the systematic evolution of ligands by exponential enrichment (SELEX) process, the derived aptamers are selected against various targets, including cells, microorganisms, proteins, and chemical compounds [66]. Compared with the aforementioned ligands (HBGA, PGM and mAbs), much less research has been conducted on utilizing aptamers for HuNoV detection. AG3, an aptamer selected for murine norovirus (MNV) through SELEX by Giamberardino et al. [67], can bind with HuNoV GII.3 capsids. An electrochemical biosensor made of gold nanoparticles-modified screen-printed carbon electrode coupled with the thiolated aptamer AG3 could achieve a LOD of around 180 virus particles of MNV, though the selectivity was not very high and non-specific binding occurred [67]. Similarly, a study conducted by Kitajima et al. [68] utilized an AG3 aptamer to develop a miniaturized and potentially portable electrochemical biosensor for detection of MNV. More recently, by using the AG3 aptamer, Weerathunge et al. [69] developed a rapid (10 min) and ultrasensitive colorimetric NanoZyme aptasensor for the detection of MNV with an experimental LOD of 200 viruses/mL and calculated LOD of 30 viruses/mL. Specifically, gold nanoparticles (AuNPs) that have enzyme-mimic catalytic activity (i.e., NanoZyme activity) were immobilized with the AG3 aptamer (Kd of 18.5 nM) for MNV detection. The NanoZyme activity of AuNPs, capable of converting 3,3′,5,5′-tetramethylbenzidine substrates to a blue colored product, was lost upon binding with aptamers, but the activity resumed upon binding with the target (MNV). The Kd of AG3 aptamer to MNV is ~10^−12^ M, therefore, upon the presence of MNV, aptamer would detach from AuNPs and bind with MNV. An advantage of this method is that the AuNPs-functionalized aptasensor is stable in solution, thus may be suitable for storage or transportation and point-of-care devices.

Similar to mAbs, currently identified aptamers do not target all genotypes of HuNoV [18]. In a study by Escudero-Abarca et al. [18], aptamers selected for GII.2 HuNoV could bind to 13 of the 14 VLPs tested, especially to GII.2 and GII.4 VLP. By using aptamer 25, aptamer magnetic capture coupled with reverse transcription quantitative polymerase chain reaction (RT-qPCR) method could detect GII.4 from artificially contaminated lettuce with a LOD of 10 genomic copies (gc) per 3 g lettuce and a capture efficiency of 2.5%~36%. For partially purified stool specimens collected from outbreaks, enzyme-linked aptamer sorbent assay (ELASA) assays using aptamer 25 were able to detect GI.1, GII.1, GII.2, GII.3, and GII.4 HuNoV, but not strains of GI.6 or GII.7. In the exclusivity study, aptamer 25 has been shown to have a significantly higher binding affinity to GII.2 VLPs than to hepatitis A virus (HAV) or poliovirus via ELASA test [18]. Using the 6-carboxyfluorescein labeled aptamer with the same DNA library as that described by Escudero-Abarca et al. [18], Weng and Neethirajan [70] developed a paper-based microfluidic device using graphene oxide as the fluorescence quencher for determination of GII NoV VLPs with a LOD of 3.3 ng center dot/mL.

Aptamers selected against the capsid protruding (P) domain of a HuNoV GII.4 strain have been developed in a more recent study by Moore et al. [71]. Among all the aptamers, aptamer M6-2 had the broadest reactivity, with low to moderate binding affinity to all of the 14 VLPs tested and could be used to detect GII.4 NoV from partially purified stool samples, by using ELASA and aptamer magnetic capture together with RT-qPCR. Though similar broad reactivity and signal/noise ratio were achieved when compared to the study by Escudero-Abarca et al. [18], the aptamer M6-2 displayed a capture efficiency of 4.88–6.79 log_10_ gc of virus input, which was lower than the capture efficiency of aptamer 25, due to the lower number of counter-SELEX rounds performed in generating M6-2 [71]. In addition, aptamers targeting NoV P domain had also been selected by Schilling et al. [72], with a Kd of 17.42 nM. However, the aptamers did not bind with the P-domain in the presence of food matrix (such as frozen strawberries, lettuce, whole oyster, and oyster digestive diverticula). Matrix-related inhibition and non-specific binding remain challenges for the utilization of aptamers in complex matrices.

### 2.2. Immune Electron Microscopy (IEM)

In 1968, the outbreak of a winter vomiting disease that occurred in an elementary school in Norwalk, Ohio brought an unknown virus into the light of researchers [73]. The prototype norovirus, also referred to as the Norwalk virus, was discovered in 1972 via the immune electron microscopy method [74]. Despite the fact that electron microscopy (EM) could visualize the shape and size of viruses, the morphology of HuNoV was very difficult to differentiate from other small round structured viruses (e.g., sapovirus) through the use of this method (which has a LOD of around 10^6^ viral particles per ml of stool) [75]. Therefore, an IEM method, which was based on electron microscopy but incorporated the use of antibodies to precipitate the virus from clarified stool filtrates, was applied to aid in the identification of non-cultivatable HuNoVs. Though EM and IEM were able to detect viral particles in a short time period (15 min), several disadvantages limited their application in the diagnostics of HuNoV; including high cost, low sensitivity and specificity, laborious operation and requirement of trained personnel. Due to these limitations, electron microscopy-based methods are largely only performed for analytical purposes and not detection.

### 2.3. Immunoassays

Immunoassays could be applied to detect the presence or concentration of protein analytes (antibody or antigen), based on the incorporation of specific ligands (antigen or antibody). They can be categorized into several types, based on the labelling methods used to detect or quantify the analytes. In the case of HuNoV detection, radio-immunoassays (RIA) with the label of radioisotopes, enzyme immunoassays (EIA) with enzyme labelling, immune adherence hemagglutination assay (IAHA), and immuno-chromatography (ICG) tests will be discussed in detail. In general, immunoassays have the advantage of portability, ease of operation, and rapid results; however, these assays generally tend to lack analytical sensitivity, specificity, and are limited in their ability to subtype.

#### 2.3.1. Immune Adherence Hemagglutination Assay (IAHA)

After the successful application of IAHA tests to the measurement of some animal viruses (herpes simplex virus, simian virus 40, adeno- and enteroviruses) and their antibodies in 1966 [76], this method has been investigated and applied in the detection of other viruses as well as antibody responses to them. For example, an IAHA test conducted by Kapikian et al. [77] was applied for the detection of antibody against Norwalk virus from acute epidemic nonbacterial gastroenteritis, by using purified viruses from stool as an antigen. Around the late 1970s, IAHA tests began to replace EM and IEM for the detection of NoVs in clinical samples [78]. IAHA used the agglutination of human erythrocytes with the antigen-antibody complex to study HuNoV sero-prevalence. Though this method is rapid, simple, and inexpensive, its inability to differentiate immunoglobulin subclasses as well as the problem of the virus agglutinating red blood cells inhibits its application. In addition, the method is not suitable for direct NoV detection in fecal samples [79,80].

#### 2.3.2. Radio-Immunoassays (RIA)

Ultimately, IAHA was replaced by RIA, which used radio-labelled immunoglobulin G (IgG) to detect NoV antigen or antibody. RIA is often conducted in a microtiter format and could be used to detect viral antigens (or antibodies) by involving a competitive binding between unlabeled and radioisotope (^125^I), labeled corresponding antibodies (or antigens). In 1978, a study by Greenberg et al. [81] using a microtiter solid-phase RIA successfully detected Norwalk virus (from stool samples) and its antibody with a much higher sensitivity than IAHA assay. When utilized for the detection of NoV antigen in stool samples, the IgG which was purified from a convalescent serum of a NoV infected patient was used as an antibody and radiolabeled. The radiolabeled IgG was then added to competitively bind with NoV antigen against unlabeled IgG. A reduction of 50% or more radioactivity defines the presence of the viral antigen.

The blocking RIA assay for NoV antibody detection was 10 to 200 folds more sensitive than IAHA and required much less antigen [82]. There have been a number of studies however, where the RIA assay was not able to detect any NoV strains [83]. Since both IAHA and RIA methods require reagents from human volunteers, their applications for the detection of HuNoV are considered to be limited [82].

#### 2.3.3. Enzyme Immunoassays (EIA)

Enzyme immunoassays (EIA) incorporate specific viral antibodies (or antigens) for the detection of viral antigens (or antibodies). EIA is similar to RIA, but utilizes enzymes rather than radioisotopes for detection. Aside from not having the potential of being exposed to radioisotopes, EIA has several advantages when compared to RIA, which includes the increased stability of reagents used, decreased assay time, and simpler equipment operation. Specifically, the radioisotope labelled antibody only has several days to weeks of shelf-life, while the antibody labelled by the enzyme or biotin has a shelf-life of several months. In addition, the assay time can be reduced by several days when compared to RIA [82]. The use of colorimetric and bioluminescent EIA methods for HuNoV detection will be discussed here in detail.

In the 1990s, the cloning of NoV capsid protein enabled the production of NoV VLPs. These were produced from baculovirus recombinants that contained NoV VP1. In particular, NoV VLPs were shown to be antigenically and morphologically similar to native NoVs, and thus have been widely used for the structural studies of NoV [84]. The use of VLPs has also allowed the detection of viral antibodies [85].

Cloned NoV capsid protein can also be used to induce antibodies against NoVs, which can then be applied in enzyme-linked immunosorbent assay (ELISA) for detection. A number of commercial ELISA kits are available for NoV detection (both GI and GII). The two most commonly used kits are IDEIA Norovirus (Oxoid Ltd., Hampshire, United Kingdom; two generations available) and RIDASCREEN Norovirus (R-Biopharm, Darmstadt, Germany; three generations available). There are wide ranges of reported sensitivity and specificity for these kits. The sensitivities for IDEIA and RIDASCREEN Norovirus range from 38.0% to 78.9% and 31.6% to 92.0%, respectively, while their specificities range from 85.0% to 100.0% and 65.3% to 100.0%, respectively [86]. Several factors contribute to the different performance of commercial EIA kits in terms of detection of norovirus outbreaks. These include the viral titer and viral genotypes present in clinical samples (since the antibodies in EIA kits have varied affinities to different NoV genotypes), specimen collection (the time collected after symptoms onset), infection extent (outbreak or sporadic), and patient demographics (pediatric or adults) [86].

Compared to the 2nd generation IDEIA ELISA test, the 3rd generation RIDASCREEN ELISA has much higher sensitivity for the detection of NoVs. According to a comparison study of these two kits by Kirby et al. [87], the sensitivity of the 3rd generation RIDASCREEN was 63% and the specificity was more than 98%, while the sensitivity of IDEIA was only 45%. Due to its combination of several cross-reactive monoclonal and polyclonal antibodies, RIDASCREEN has the capability to detect some HuNoV genotypes. Despite these advantages, RIDASCREEN tests generally have low sensitivity. In particular, samples from sporadic NoV cases with GI and mixed infections (GI/GII) were unlikely to be detected by the kit [88]. With a LOD of 10^4^–10^6^ viral particles/mL of stool by ELISA, it is not likely to be sensitive enough for detection of NoVs in food or environmental samples [78]. Moreover, since HuNoVs exhibit at least 142 dissimilar antigenic behaviors, the development of an EIA that is cross-reactive to all HuNoV genotypes is difficult [11].

Considered to be more than just a routine ELISA test, a bioluminescent enzyme immunoassay (BLEIA) conducted by Sakamaki et al. [89], was reported to be able to detect HuNoV VLPs, including 6 GI and 8 GII. This assay had a good reproducibility, with a turnaround time of 46 min and a throughput of 120 tests/h. A similar BLEIA method by Shigemoto et al. [90] could detect 3 NoV GI genotypes and 10 GII genotypes from fecal samples with a LOD of 10^6^ gc per gram of stool and below and a sensitivity of 96.3%. Similar results were observed in the study by Suzuki et al. [91], with higher sensitivity (93.1%), specificity (100%) and detection rate (95.7%) of BLEIA method compared to the reverse transcription loop-mediated isothermal amplification (RT-LAMP) method, for the detection of HuNoVs from fecal samples. It was found in these studies that BLEIA assays did not show cross-reactivity towards bacteria or other enteric viruses and the sensitivity was around 10^5^–10^6^ copies/g stool samples, which is roughly comparable to that observed for ELISA.

#### 2.3.4. Immuno-Chromatography (ICG)

Aside from the use of commercial ELISA tests, a number of commercial ICG lateral flow assay (LFA) kits are also available for NoV detection (both GI and GII). ICG kits have a number of advantages, including a short timeline (result achieved within 15~30 min), long shelf life (12~24 months), ease of use, relatively low cost (not requiring specific laboratory equipment), and relative ease in manufacturing. Such kits include Ridaquick Norovirus (R-Biopharm, Darmstadt, Germany), SD Bioline Norovirus (Standard Diagnostics, Inc., Kyonggi-do, South Korea), ImmunoCardSTAT!^®^ Norovirus (Meridian Bioscience Europe, Nice, France), and NOROTOP^®^ (ALL.DIAG SA, Strasbourg, France). A study comparing these four kits concluded that the clinical sensitivity for the detection of GII.4 stool samples is 78%, 59%, 61%, and 67%, respectively [92]. The ICG kits, including two commonly used kits (Ridaquick and SD Bioline Norovirus), are shown to have a wide variability of performance, due to similar reasons as those described for ELISA kits, and are related to challenges with the antibodies used. Specifically, the sensitivities for Ridaquick and SD Bioline Norovirus range from 17.0% to 83.0% and 23.0% to 92.0%, respectively, while the specificities for Ridaquick and SD Bioline Norovirus range from 87.5% to 100.0% and 99.7% to 100.0%, respectively [86]. Due to the wide variation of the sensitivity and specificity, more sensitive molecular detection methods are recommended for samples that have negative results from the ELISA or ICG kits [86].

The detection of the Ridaquick Norovirus ICG kit is based on incorporating gold-labeled anti-NoV antibodies and biotinylated anti-NoV antibodies. A streptavidin test line could capture the sandwich complex, while the unbound complex would migrate to the control line. The high LOD of traditional LFA by using blue latex or AuNPs could be improved by pre-concentration or the use of other reporter particles (e.g., enzyme labeled particles, photoluminescent particles, and phage nanoparticles). An example is the LFA study conducted by Hagström et al. [93], who incorporated M13 phage nanoparticles as reporters, and an antibody pair to detect GI.1 Norwalk VLPs. This assay gave a LOD of 10^7^ VLPs/mL, which was 100-fold more sensitive than a conventional gold nanoparticle LFA test.

#### 2.3.5. Western Blot

The western blot (also called protein immunoblot) assay has been widely used for the analysis of proteins in tissue homogenates or extracts and its use is composed of several steps. First, the proteins of the analytes are separated by sodium dodecyl sulfate–polyacrylamide gel electrophoresis (SDS-PAGE), according to their molecular weight. Then the protein bands are transferred to a nitrocellulose membrane, followed by blocking and the addition of a specific primary antibody and enzyme labeled secondary antibody. The step of gel electrophoresis reduces the probability of the cross-reactivity of antibodies. Therefore, western blot seldom gives false positive results and it is the most commonly used technique to confirm the positive results from ELISA tests [94]. However, compared to ELISA tests, western blot is more difficult to perform and it requires higher skill.

Western blot has been used for the detection of NoV GII.4 VLPs. The VLPs are separated by SDS-PAGE, then IgG and horseradish peroxidase (HRP)-conjugated goat anti-mouse IgG were incorporated to capture and detect VLPs. The immunogenicities of VLPs and P particles were studied by western blot, and the results showed that VLPs had higher immunogenicity than P particles [95].

Western blot has also been used in the study of mapping NoV antibodies. By testing the interaction of mAbs with the deletion mutants of GII.4 VLP, the binding sites of the antibodies could be identified [55]. Finally, Western blot has also been applied in the evaluation of antibody response after viral infection, by using a crude small round-structured virus sample as an antigen. The major viral structural protein could also be determined by Western blot [96].

## 3. Nucleic Acid-Based Techniques

The sequencing of the NoV genome contributed to the development of nucleic acid based assays for HuNoV detection [97]. During the mid-1990s, the first conventional RT-PCR targeting a relatively conserved small region of the RNA polymerase gene in open reading frame (ORF)1 was used for the detection of NoV [11]. Since then, more real-time rapid and sensitive assays, including TaqMan RT-PCR, SYBR Green RT-PCR and isothermal amplification assays have been developed for NoV. By targeting the detection of a conserved genome region at the ORF1-ORF2 junction, real-time nucleic acid based methods are more sensitive and broadly reactive than antigen detection assays [78].

### 3.1. Real-Time Reverse Transcription-Polymerase Chain Reaction (RT-PCR)

RT-PCR is currently considered to be the gold standard for detecting HuNoVs in clinical samples, food, water, or environmental samples. It is also recommended by the International Organization for Standardization (ISO) in technical specification documents ISO/TS 15216 to be applied for the quantitative and qualitative detection of NoV in food and water [98,99]. However, problems associated with false positives or inhibition can occur in RT-qPCR assays. It is also known that some PCR inhibitors are present within samples such as food and stool [78]. Therefore, a good elution/extraction, concentration and purification method before viral nucleic acid testing is very important. In addition, the RT-PCR method is reagent-intensive, relatively slow (40 min~3 h) and generally requires connection to an electrical grid [100]; though microfluidics-based techniques have been reported that address these. Further, this method cannot differentiate infectious from non-infectious HuNoVs. One of the additional challenges with RT-qPCR and other nucleic acid-based techniques is the high diversity of norovirus strain sequences coupled with the comparatively short length of the genome. However, numerous broadly reactive primers (generally limited to genogroup-level reactivity) have been reported, with most targeting the ORF1-ORF2 junction of the genome [11].

Multiplexed RT-qPCR can be used in the simultaneous detection of GI and GII strains or GI, GII, and GIV strains of HuNoVs [101,102,103]. Several FDA-cleared multiplex molecular tests are commercially available, including the xTAG(^®^) Gastrointestinal Pathogen Panel (GPP), FilmArray™ and Verigene^®^ Enteric Pathogens Nucleic Acid Test, and can achieve results within several hours [11]. For example, the xTAG(^®^) GPP (Luminex Corporation, Toronto, Canada) could detect 15 gastrointestinal pathogens (including viruses, bacteria and parasites) within 5 h for >90% of pathogens. It has an overall sensitivity and specificity of 94.3% and 98.5%, respectively, according to a test of 901 stool specimens from both children and adults [104]. There are also several molecular assays as in vitro diagnostic devices approved by Europe with CE Marking certificate, including AndiaTee Norovirus real RT-PCR kit, RealStar Norovirus RT-PCR kit, Xpert Norovirus kit and Allplex™ Gastrointestinal Panel Assays [105].

### 3.2. Isothermal Amplification Methods

Although still considered one of the most sensitive and gold standard techniques, real-time RT-PCR has the limitations of lack of portability, takes ≥ 40 min to get a result, and is sensitive to inhibitors. Due to this fact, numerous isothermal amplification techniques for the detection of NoV have been reported; specifically the nucleic acid sequence-based amplification (NASBA) [106], loop-mediated isothermal amplification (LAMP) [107] and recombinase polymerase amplification (RPA) [108]. For instance, Moore et al. [106] found that NASBA is an alternative highly sensitive and specific method for NoV detection. Specifically, the sensitivity and specificity of NASBA as compared to that of RT-PCR was 100% and 80%, respectively [106]. In addition, Moore and Jaykus [108] developed a rapid (<30 min) RPA assay for the detection of HuNoV RNA from outbreak samples, with a LOD of ~3.4 log_10_ gc. Jeon et al. [109] developed a one-step RT-LAMP assay for the detection of GI and GII HuNoV and found that one step RT-LAMP was 10~100 times more sensitive than real time RT-PCR. A colorimetric RT-LAMP assay with a metal ion-binding indicator dye (hydroxynaphthol blue dye) was reported to have a sensitivity of 10^3^ gc per reaction and a detection rate of 94.83% in detection of GII NoV [110]. Different to the conventional RT-LAMP assay, the test result could be visually observed. Positive samples showed a color change from violet to sky blue. The turbidity of the reaction could be measured with a turbidimeter and the value could be compared against a cutoff value [110].

### 3.3. Nucleic Acid Based Methods to Assess HuNoV Infectivity

Currently, there is no standard method to assess HuNoV infectivity. Although some advanced nucleic acid based assays have been explored to assess infectivity [111], each method has their own limitations or shortcomings. Due to the fact that amplification-based techniques focus only on detection of the presence of nucleic acid, they will not be able to discriminate between nucleic acid associated with infectious virus versus that which is not. A few of the methods to better estimate viral infectivity will be discussed; including long template RT-PCR, RT-PCR with intercalator, enzymatic pretreatment, and receptor binding-pretreatment. For all of the mentioned techniques, none are able to completely able to predict the signal observed with cultivable surrogates in inactivation studies, though many do remove a notable portion of noninfectious particles.

Long template RT-PCR, which uses a long range of viral sequences for amplification, has been investigated by several researchers due to its potential for assessing the integrity of the viral genome. Currently, the real time RT-qPCR test only focuses on a small conserved region to test virus RNA titer. However, it is possible that other regions of the genome are at risk of being damaged, while no RNA reduction is shown by the real time RT-qPCR test. Since viral genome regions demonstrated heterogeneous sensitivities to some inactivation treatments (e.g., UV), the damage of the genome does not follow the Poisson distribution [112]. A long template RT-PCR, which allows for the amplification of a near full-length genome of NoV (7295~7360 nucleotides), had been used for the determination of the near complete genome sequence of two GII clinical isolates [113]. However, long template RT-PCR has low efficiency, sensitivity, and takes a long time, due to the amplification of such a large target [112]. In addition, Seo et al. [114] showed that long template RT-PCR gave a significantly underestimated reduction of infectious NoV surrogates (MNV and MS2) after heat, salt or pH treatments; indicating that noninfectious RNA was amplified. This is likely due to the fact that this method alone does not account for RNA encapsidated in fatally damaged or mutated capsids.

Nucleic acid intercalators, including photoactivatable dye propidium monoazide (PMA) and ethidium monoazide (EMA), have been used to assess the viability of bacterial cells (including Escherichia coli and Bacillus subtilis) and viruses (including bacteriophage MS2 and HAV) [115,116]. Upon excitation by visible light (high power halogen lamps or specific LED devices), the azide group in the dye converts to a nitrene radical, which covalently binds to nucleic acid and results in stable products non-amplifiable by PCR. The intercalators are impermeable to intact cell membrane or viral capsids, and only crosslink with nucleic acids from damaged cell membranes or viral capsids. By incorporating a PMA pre-treatment prior to RT-PCR, the detection of noninfectious viruses could be eliminated to some extent [115]. Although this method has been used to selectively differentiate infectious and noninfectious viruses, under some circumstances, it can be unreliable due to its inconsistency in performance. For example, RT-PCR with PMA pre-treatment has successfully differentiated intact viral particles (MNV-1 and NV) from naked viral RNA, but it is unable to do so between infectious and noninfectious NV (by any treatment) [117]. In addition, it was shown that EMA-coupled RT-qPCR underestimated GII.4 HuNoV reduction by cold plasma [118]. Improved versions of PMA and EMA (PMAxx and PEMAX) are commercially available, and PMAxx has been shown to have a better performance than conventional photoactivatable dyes (PMA and EMA) in assessing NoV infectivity. According to a study conducted by Randazzo et al. [117], pretreatment with PMAxx and surfactant (Triton X-100) was able to discriminate infectious and thermally inactivated NoV, with the latter having ~1.4 to > 2 log reductions of RT-PCR signals. In addition, this pretreatment step could be easily incorporated into the ISO 15216 method for detection of HuNoV or HAV in food and water samples [117]. However, in theory, this method would not account for particles with damaged or fatally mutated P domain, for which icosahedral contacts maintain intact, which is a possibility observed for this and enzymatic pretreatment (below) [119]. More research needs to be conducted to show the efficacy of this method in assessing NoV infectivity.

Enzymatic pretreatment (ET), combined with a nucleic acid-based detection method, has also been proposed as a potential assay to detect infectious HuNoVs. Enzyme pre-treatment (a combination of proteinase K and RNase A) (one step) was introduced by Nuanualsuwan and Cliver [120], which could reduce false-positive signals by degrading damaged viral capsids as well as naked viral RNA. Though no false positive results were observed when ET combined with the real time NASBA method were applied for detection of heat treated feline calicivirus (FCV) and HuNoV according to Lamhoujeb et al. [9], further investigation of how accurate this method is for the detection of other virus strains and inactivation treatments is needed. In addition, although RNase treatment can degrade viral RNA with damaged viral capsids (not intact viral particles) and is very stable, the presence of small low molecular weight RNA fragments or ribonucleoproteins (RNPs) can be detected by RT-PCR. Moreover, RNase resistant RNPs can be released from HuNoV viral particles under heat inactivation treatments (e.g., 45 °C for 1.5 min) [112]. The partially damaged capsid may also render the residual protection of RNA. Furthermore, the combination of proteinase K and RNase A is very difficult to control, since the RNase could be degraded by proteinase K, which needs to be stabilized in 1 mM calcium ions [112]. Though separating ET by adding proteinase K and RNase A sequentially (two steps) were reported to be more sensitive at reducing false-positive signals than one step ET, neither was able to completely remove false-positive signals [121].

Among all the proposed nucleic acid-based methods for infectious HuNoV detection, the use of a ligand binding step prior to detection by RT-qPCR is the most widely used and has been shown to be both a rapid and sensitive technique for removing noninfectious viral particles. Magnetic beads can be conjugated with different ligands (i.e., PGM, HBGA, antibodies or aptamers) for the specific capture of HuNoV from samples. Cannon and Vinjé [122] utilized a binding step with magnetic beads conjugated with H type 1 HBGA followed by RT-PCR and were able to detect 30~300 gc of the Norwalk virus from environmental waters. The use of PGM-MB combined with the RT-qPCR assay was proposed by Dancho et al. [123] as a potential method to discriminate infectious from non-infectious NoVs, since a reduced binding of NoVs to PGM-MB was observed after inactivation treatments (UV, thermal or high pressure). However, the efficacy of this method varies, depending on the HuNoVs strains and inactivation treatments, as shown by Afolayan et al. [124], where complete elimination of RT-PCR signals was achieved for both GI.1 and GII.4 when inactivated by the levulinic acid plus sodium dodecyl sulfate treatment but not after heat treatment (99 °C for 5 min). It is believed that non-infectious viruses could also be detected in the RT-qPCR assay with the PGM-MB pre-treatment, since partially damaged viral capsids may have a portion that is able to both protect viral RNA from RNase degradation, but also bind to PGM after inactivation treatments [124,125]. Aside from HBGA and PGM, antibodies have also been conjugated to magnetic beads to capture NoV from contaminated samples and combined with RT-qPCR for detection, which would still likely result in removal of naked RNA and matrix-associated inhibitors but would likely capture damaged capsids. Interestingly, Moore et al. [119] demonstrated that aptamer M6-2 displayed similar dependence on intact viral capsids for binding as HBGAs, suggesting that aptamer M6-2 could be utilized in a binding step to detect intact viral particles. Park et al. [126] successfully detected NoV from artificially contaminated strawberries with a LOD of 3 ~ 7 RT-PCR units and a recovery rate of 14% ~ 30%. The use of magnetic bead separation with RT-qPCR has several advantages, including concentrating viruses and removing inhibitors from the food matrix. By conjugating with antibodies, PGM or HBGA, the magnetic separation method could only bind to viral capsid (not naked viral RNA), which could eliminate the detection of inactivated viruses with a naked viral RNA. However, these methods are also unable to completely differentiate infectious from noninfectious viruses with fatally mutated or damaged genomes, and the specificity of this method is strongly dependent on that of the capture ligand used. The fact that broadly reactive ligands are unavailable also hampers the application of this technique in the detection of various HuNoV strains [127].

## 4. Biosensors

Biosensors have received much interest over the past decade and have been increasingly studied for NoV detection. A biosensor is an analytical device that converts a biological response into an electrical signal by integrating a biologically active element with an appropriate physicochemical transducer or transducing system. Biosensors are primarily grouped into optical, electrochemical, piezoelectrical, thermal, magnetic and micromechanical biosensors, according to the signal transducers used [128]. A summary of the available biosensor-based methods for the detection of NoV and its surrogates is shown in Table 3.

### 4.1. Optical Biosensors

Optical biosensors have several subclasses based on the measurement of adsorption, reflection, refractive index, Raman, infrared (IR), fluorescence, chemiluminescence, and energy transfer [161,162]. A number of optical biosensors, including surface-enhanced Raman spectroscopy (SERS), surface plasmon resonance (SPR), and evanescent wave-based biosensors, have been applied in virus detection. These detection methods are often rapid, label-free and have high sensitivity or specificity. Unlike ELISA-based techniques, optical biosensors require minimal sample preparation and can detect at near real-time.

#### 4.1.1. Surface Enhanced Raman Scattering (SERS)

Surface enhanced Raman scattering, or surface enhanced Raman spectroscopy (SERS), is an optical detection technique that enhances Raman scattering of molecules adsorbed on or close to metal nanostructures. SERS is a rapid method that has been used frequently for pathogen detection and disease diagnosis. It has been applied for the detection and differentiation of a number of viruses, including adenovirus [130] and rotavirus [163]. The SERS technique can provide a ‘fingerprint’ spectrum for each sample, giving information on structure and constituents. Compared with other detection methods, SERS has the benefit of less volume input, high sensitivity, and high specificity. Furthermore, it can also be used as a qualitative or quantitative detection method for virus samples. Quantification of virus titers is possible if proportional relationships are achieved between SERS peak intensities and sample concentrations [164].

When the SERS technique is applied for detection purposes, the two formats designated as intrinsic and extrinsic are usually used. For intrinsic SERS (also known as label-free) detection methods, the spectrum is obtained from the target molecule without the use of extrinsic Raman labels (ERLs), while extrinsic SERS uses ERLs. Among the two, the intrinsic detection method is considered to be more preferable as the introduction of ligands or Raman labels may bring in uncertainties [165].

**Figure 1:**
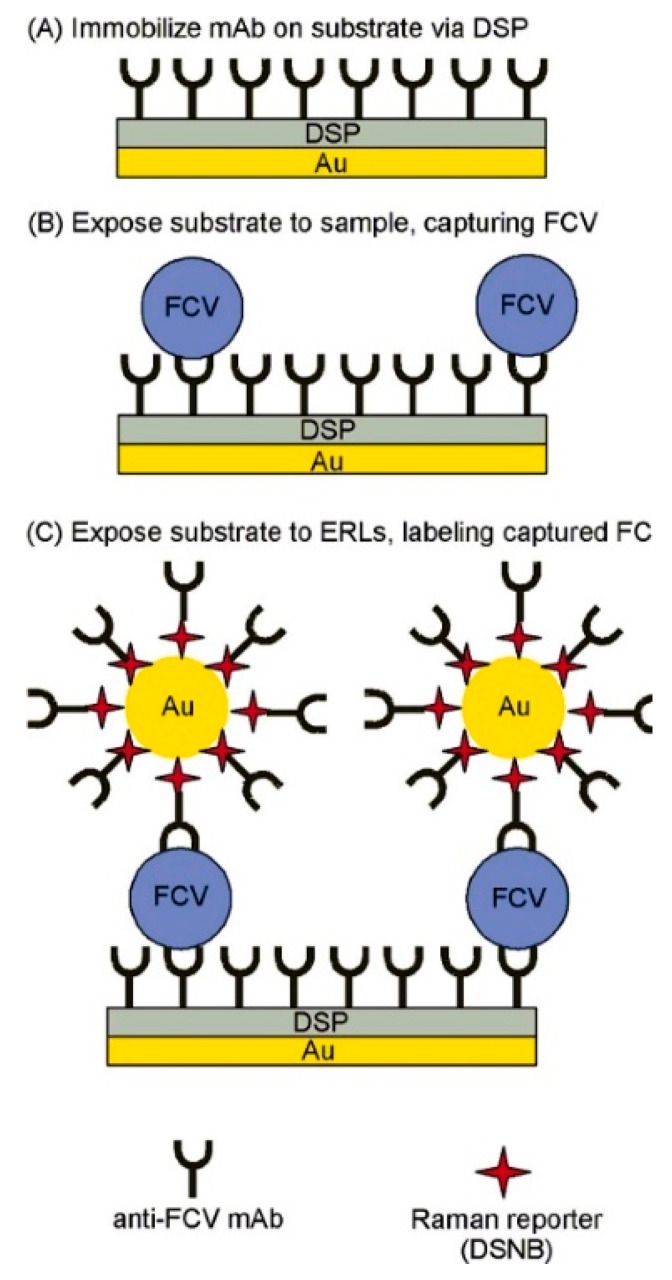
Detection of feline calicivirus (FCV) by extrinsic surface enhanced Raman scattering (SERS) (reprinted from Driskell et al. [129] with permission from the publisher).

SERS has also been applied towards the detection and differentiation of HuNoV surrogates FCV and MNV. Driskell et al. [129] successfully utilized SERS to detect FCV. This was achieved through a sandwich structure composed of a mAb immobilized gold substrate to capture FCV from cell culture and an ERL, as depicted in Figure 1. Specifically, the ERL consisted of mAb conjugated AuNPs, linked with a Raman reporter molecule 5,5′-dithiobis(succinimidyl-2-nitrobenzoate). A LOD of 10^6^ viruses/mL was achieved for FCV with this method [129]. MNV detection using SERS has been demonstrated by Fan et al. [130], whom successfully detected and differentiated the norovirus strain MNV-4 from other virus strains (i.e., adenovirus and rotavirus), based on their intrinsic SERS spectrum. Results also showed that purified MNV-4 was differentiable from unpurified MNV-4 (containing Vero cell lysate) and control (Vero cell lysate). The bands from MNV-4 at 744 and 937 cm^−1^ corresponded to adenine and C−COO− stretch, respectively.

Despite these achievements in the detection of HuNoV surrogates, SERS has not yet been applied in the detection and differentiation of HuNoVs. A major obstacle lies in the fact that HuNoV clinical samples are collected from feces of infected individuals, with a large variation in sample impurities. Therefore, some pretreatment of the samples may be required to selectively capture HuNoV from the feces or remove impurities. Additionally, the equipment cost for SERS is high (approximately $15,000), and incubation time may be long, though miniaturized instruments are available [141].

#### 4.1.2. Surface Plasmon Resonance (SPR)

Surface plasmon resonance (SPR) is an oscillating phenomenon that occurs in thin conducting films placed at the interface between two media of different refractive indices. When a binding event occurs, changes in mass near the sensor chip surface causes a change in the refractive index. SPR is a highly sensitive, quantitative and rapid label-free technique used to study the binding affinity between biomolecules. It has been widely used for virus detection, including Epstein–Barr virus, hepatitis B virus (HBV) and human immunodeficiency virus type 1 (HIV-1) as well as viral protein detection, such as HA proteins in the influenza virus (serotypes H1N1 and H3N2) [36].

SPR analysis has been utilized to study the binding affinity and kinetics of ligands with NoVs. Specifically, Kou et al. [16] reported the strong binding of multiple mAbs, with a Kd value of <10 nM with NoV VLPs. In addition, SPR could be used for the identification of a broadly reactive scFv (HJT-R3-A9), that could bind with multiple NoV VLPs [166]. Aside from antibody binding studies, SPR has also been applied to the study of the interaction between human milk glycan and NoV VLPs (strains VA387 and Norwalk), as well as ABH antigens binding to VLPs [41,167]. Shang et al. [41] utilized SPR imaging to show that human milk glycan motif specifically binds to NoV and competitively inhibits viral capsid binding to host receptors. By SPR, Hurwitz et al. [57] identified two scFvs (NJT-R3-A2 and NJT-R3-A3) that could bind to GI.1 and GI.7 VLPs, with Kd values of 27 nM and 49 nM, respectively. Similarly, Kim et al. [137] demonstrated that concanavalin A (Con A) can bind with HuNoV (GII.4), with the metal coordination region of Con A identified as a major region of the interaction. Specifically, the Kd values determined for native Con A and metal coordinating region-mutated Con A by SPR were 71.5 ± 6.7 nM and 22.0 ± 17.0 μM, respectively. By using Con A-immobilized polyacrylate beads, HuNoV (GI and GII) could be rapidly detected in 15 min with 90% recovery over a wide range of pH values (pH 3.0~10.0) [137].

**Figure 2:**
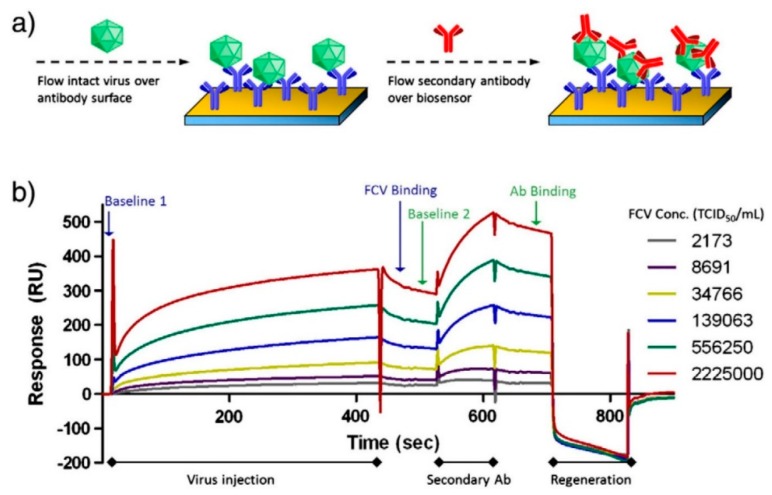
Detection of feline calicivirus (FCV) via a surface plasmon resonance (SPR) assay. (**a**) The diagram of the detection assay. (**b**) The sensorgram (reference flow cell subtracted) for FCV (with different concentrations) and secondary antibody (Ab). A greater response (RU) achieved when FCV and secondary Ab binds (reprinted from Yakes et al. [136] with permission from the publisher).

Furthermore, a localized SPR-based immunofluorescence nanobiosensor developed by Takemura et al. [132] was able to achieve a LOD of 0.4 pg/mL of NoV VLPs, by incorporating antibody conjugated AuNPs and quantum dots (QDs). A similar study of an SPR-assisted fluorescence sensor developed by Ashiba et al. [138] using QDs, with incorporation of mAb (12A11) and pAbs as capture and detection antibody against NoV VLPs, had a LOD of 0.01 ng/mL of NoV VLPs. Aside from application of detection of NoV VLPs, SPR had also been used for FCV detection [136], as shown in Figure 2. Via immobilization of an anti-FCV antibody onto a SPR chip surface, this assay was rapid (within 15 min) and had a low LOD of around 10^4^ TCID_50_ FCV/mL for purified cell culture lysates. Moreover, this assay had a potential of detecting FCV from spiked oyster matrices if an extraction procedure was applied [136]. However, SPR has several limitations. One limitation is that the native configuration of ligands may change upon immobilization on sensor surface, which may hinder the binding of analytes [168]. In addition, non-specific binding needs to be carefully controlled when using SPR for the biosensing of molecular interactions [168].

#### 4.1.3. Other Optical Biosensor Methods

**Figure 3:**
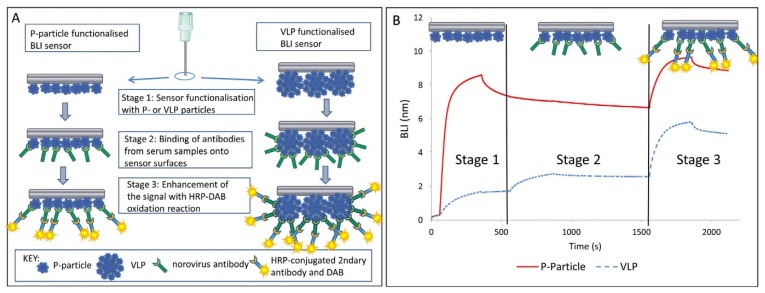
Detection of norovirus-like particles (VLPs) using the biolayer interferometry (BLI) based biosensor method. (**A**) Schematic representation of the BLI detection method utilizing NoV VLP or P-particles functionalized sensor. (**B**) Sensorgrams of a NoV-positive serum sample. The red and blue curve correspond to the NoV P-particle and VLP functionalized sensor, respectively. (Reprinted from Auer et al. [145] with permission from the publisher).

Aside from SERS and SPR, a number of other optical biosensor methods, including biolayer interferometry, fluorescence, bioluminescence and colorimetric based biosensors have also been shown to successfully detect NoV. For example, a label- and fluidic-free detection system based on a biolayer interferometry (BLI) biosensor has been proven to be able to detect NoV antibodies from human serum samples [145]. The scheme is depicted in Figure 3. This assay is considered rapid (10~20 min if the sensor was pre-functionalized) and could detect NoV antibodies in serum dilutions up to 1:100,000 [145]. Similarly, Auer et al. [144] used a Co (III)-NTA functionalized BLI sensor immobilized with His-tagged VLPs for the detection of NoV antibodies from human serum. The functionalization with Co(III)-NTA offers highly stable immobilization for His-tagged proteins which could withstand harsh chemical conditions. However, these two BLI based biosensor methods did not offer the direct detection of HuNoV. Future research is needed to investigate the possible application of BLI based biosensors for direct HuNoV detection.

**Figure 4:**
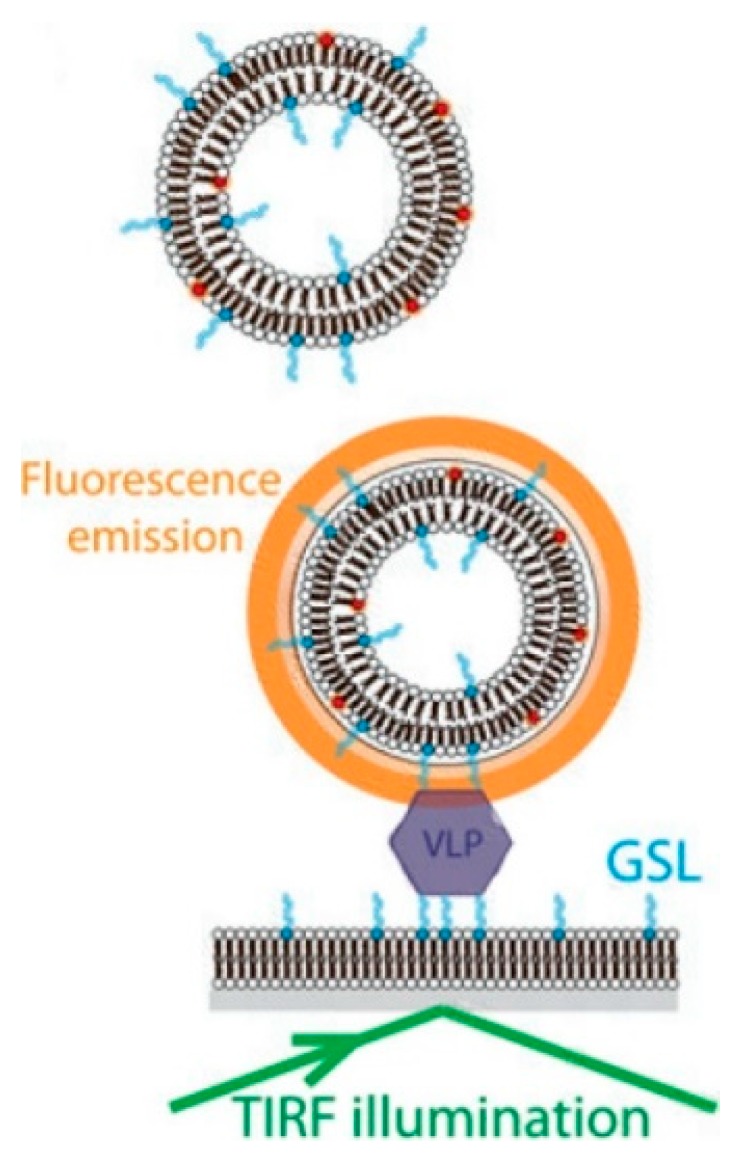
Detection of norovirus-like particles (VLPs) by total internal reflection fluorescence (TIRF). H type 1 glycosphingolipids (GSL) are used as receptors for VLPs. A GSL contained rhodamine-labeled vesicle emits fluorescence after TIRF illumination. (Reprinted from Bally et al. [140] with permission from the publisher).

A fluorescence based biosensor developed by Bally et al. [140] was able to successfully detect single NoV VLP (GII.4), by incorporating a H type 1 GSL attached lipid bilayer as a substrate support and a GSL contained rhodamine-labeled vesicle to emit a fluorescent signal after total internal reflection fluorescence (TIRF) illumination, as shown in Figure 4. This assay could achieve a LOD of 16 fM and a single-molecule sensitivity, since individual vesicles could be visualized by TIRF microscopy [140]. In another study conducted by Connelly et al. [141], a fluorescence based microfluidic biosensor was shown to be able to detect FCV with a LOD of 1.6 × 10^5^ PFU/mL within 2.5 h. Another fluorescence based biosensor developed by Zhao et al. [142] had been shown to be able to detect NV RNA with a limit of quantification of 0.005 ng/mL within 1 h. The F0F1-ATPase molecular motor biosensor was applied to detect NV RNA with a “ε-subunit antibody-streptomycin-biotin-probe” system. The fluorescence intensity change compared to the control showed that the virus was successfully captured and conjugated to the probe [142].

**Figure 5:**
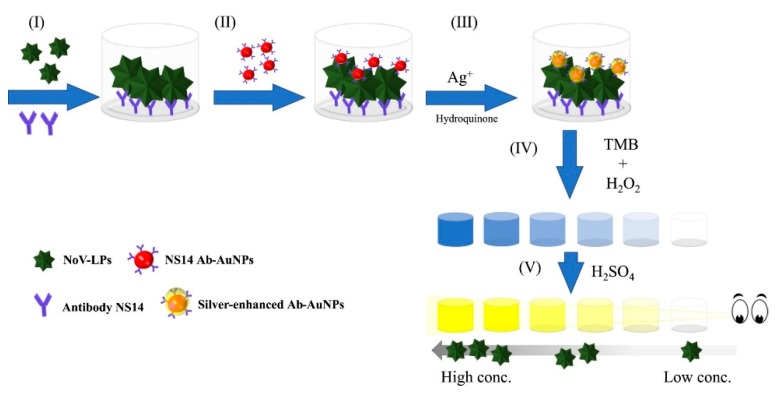
Naked-eye detection of norovirus-like particles (NoV-LPs) by a silver-enhanced nanozyme-based immunoassay (reprinted from Khoris et al. [151] with permission from the publisher).

Due to much lower background noise, chemiluminescence was described to be more sensitive than UV-vis absorbance and fluorescence [143]. Kim et al. [143] developed a chemiluminescence based biosensor by using a modified DNA aptamer for the rapid and highly specific detection of NoV GII capsid in tap water with a LOD of 80 ng/mL. In addition, colorimetric based sensors have also been used for NoV detection. For instance, Khoris et al. [151] developed a silver-enhanced nanozyme-based immunoassay for detection of NoV VLPs with naked-eye, as shown in Figure 5. The enhanced immunoassay could achieve a LOD of 10.8 pg/mL NoV VLP, as well as 13.2 and 16.3 copies/mL fecal solution (or 132 and 163 copies/g feces) for NoV GII.3 and GII.4, respectively [151]. Similarly, Ahmed et al. [169] used peroxidase-like graphene-AuNPs for visible detection of NoV VLPs with a LOD of 92.7 pg/mL. Han et al. [170] also developed a naked eye microfluidic 3D slip paper-based analytical device for the detection of GII.4 NoV from human feces with a LOD of 9.5 × 104 gc/mL. Furthermore, Lee et al. [171] developed a supersensitive sensor using a 3D total internal reflection scattering defocus microscopy, with wavelength-dependent transmission grating for the detection of NoV with a LOD of 820 yM. This method was stated to be applicable for the early stage infection of HuNoV.

### 4.2. Electrochemical Biosensors

Although optical methods have broad applications in the detection of HuNoV, several biosensors utilizing electrochemical techniques have also been successfully used in NoV detection. Electrochemical biosensors transduce a biological recognition event to electrochemical (i.e., amperometric, potentiometric or impedimetric) signals. Specifically, these have included bacteriophage MS2, HBV, adenovirus and rotavirus with a LOD of 10^3^~10^10^ viral particles/mL [172]. Electrochemical biosensors have been used for the detection of small analytes as well as large molecules. Some advantages of electrochemical detection assays were reported to be inexpensive, simple and robust [173].

**Figure 6:**
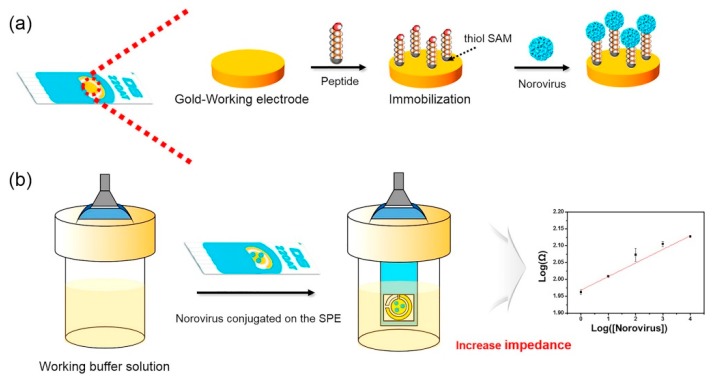
Schematic illustration for norovirus detection using an impedance electrochemical biosensor. (**a**) Peptides were immobilized as self-assembly monolayers (SAMs) on the gold-working electrode. (**b**) Working buffer solution can be used for oxidation and reduction. Dropped norovirus conjugated with the affinity peptide on the gold screen-printed electrode (SPE) and measured by electrochemical impedance spectroscopy. (Reproduced from Baek et al. [158] with permission from the publisher).

For example, a dielectrophoretic impedance measurement method developed by Nakano et al. [156] could detect 2.5 ng/mL of NoV capsid in 5 min. Compared with the immunochromatography method, this method had comparable sensitivity but required shorter time. Target viruses could be captured in the gap of microelectrodes by dielectrophoresis and the resulting conductance increase is associated with the number of viruses being trapped [156]. In another study [157], an electrochemical biosensor composed of a gold electrode conjugated with Con A could detect 60 copies/mL of NoV (GII.4) from lettuce, with a 98% selectivity within 1 h. The detection was achieved by immobilizing Con A on a gold sensor surface for the selective capture of NoV, followed by addition of NoV antibodies and alkaline phosphatase (ALP)-labeled secondary antibodies. ALP could convert 4-aminophenylphosphate monosodium salt hydrate to aminophenol, which could then be oxidized and generate current. The signal of the current was proportional to the amount of NoV captured on the sensor surface. Similarly, an electrochemical microfluidic biosensor developed by Connelly et al. [141] was able to detect FCV, with a LOD of 3.2 × 10^6^ PFU/mL. The detection was achieved by loading anti-FCV pAb-labeled Protein A superparamagnetic beads onto the device to create a capture bed, followed by the addition of pre-incubated mixture of FCV and anti-FCV mAb-electrochemical liposomes. Finally, a detergent was added to lyse the liposomes and the integrated current signal was positively correlated with virus concentration [141]. More recently, Baek et al. [158] developed an electrochemical biosensor (as shown in Figure 6) for the highly selective detection of clinical HuNoV GII.4 samples by using a NoroBP-nonFoul (FlexL)_2_ peptide assembled gold screen-printed electrode (SPE) with a LOD of 1.7 copies/mL, which is 3-fold lower than other reported methods [148,150,170]. The SPE was reported to have higher reproducibility and reliability than the conventional electrochemical sensors using non-fixed three-electrodes (i.e., working electrode, counter electrode and reference electrode).

### 4.3. Piezoelectric Biosensors

Piezoelectric mass-based biosensors have been investigated for virus detection [173]. Mass based biosensors include quartz crystal microbalance (QCM) and microcantilever arrays (mainly atomic force microscopy (AFM)). Quartz crystal microbalance with dissipation monitoring (QCM-D) has been used for real time characterization of molecular interaction with surfaces or interactions between molecules [174]. QCM has been used for the detection of a variety of viruses (e.g., HBV) [172]. AFM has been used for 3D surface topological studies of viruses and cells. It is nondestructive and noninvasive [175]. AFM has also been used for the detection of a number of viruses (e.g., HIV) [173].

Currently, mass-based biosensors are in their preliminary stage of development in terms of detecting HuNoVs. QCM-D has been used for studies in the binding of NoV VLPs with galactosylceramide and GSL [39,176]. QCM monitoring has also been studied for the detection of NoV RNA by using a Padlock probe and rolling circle amplification method [177]. Thus far, no or few studies have reported the LOD of NoV or its VLPs by the QCM-D method. By studying a sandwich-type proximity ligation assay for NoV VLP detection, Neumann et al. [178] demonstrated that a pronounced slipping effect can occur in multilayer biological systems, which could cause energy dissipation followed by mass underestimation when using QCM. Therefore, the piezoelectric mass-sensitive devices have limited applications.

AFM has also been used for NoV detection. Driskell et al. [129] reported that AFM could be used for the quantification of FCV, as signal increased with higher virus concentrations, and the results corresponded with that determined by SPR. Recently, Aybeke et al. [179] used high-speed AFM and SERS for analysis of MNV infection. AFM has also been used for imaging the integrity of NoV VLPs by a nanoindentation study [180].

## 5. Microarray

Microarrays consist of a large panel of specific probes immobilized on surfaces, which could detect virus genotypes based on solid phase probe hybridization. Microarray tests allow for simultaneous detection and subtyping of thousands of genes or target sequences within a short period of time [181]. Moreover, the rapid detection and differentiation of viruses is possible by determining the identity of the viruses directly from the detection signals [182]. As an example, a long oligonucleotide (70-mer) DNA microarray developed by Wang et al. [183] was able to detect hundreds of viruses at one time.

Over past years, microarrays have been explored for rapid detection and genotyping of NoVs. Pagotto et al. [184] at Health Canada developed an oligonucleotide array (NoroChip2.0), for simultaneously detecting and genotyping NoVs (GI and GII), through hybridization with a 917-bp RT-PCR product of NoV. Following that, Mattison et al. [165] developed the NoroChip v3.0, which has the capability of screening for over 600 interactions at one time, and had been validated in several international laboratories. However, limitations existed in the application of NoroChip v3.0 for strain typing, due to the difficulty of obtaining a long and specific amplicon from all NoV strains [185]. Another study conducted by Yu et al. [186], who optimized a custom DNA microarray (FDA_EVIR) for the detection of NoV, showed that the amplification-free detection of NoV within 250~500 copies of viral RNA could be achieved. A variation in the microarray technique was introduced by Brinkman and Fout [187], who developed a generic tag array for successful detection and genotyping of 8 NoV genotypes (GI and GII) in tap and river water samples. This was achieved through the use of RT-PCR amplicons in a single base extension reaction with labeled genotype specific probes and hybridization to a GenFlex^TM^ Tag Array. In addition, Quinones et al. [188] developed a microarray based platform for sensitive genotyping of 12 (GI/GII) HuNoV genotypes and 2 HAV genotypes from clinical samples, with a detection threshold of < 10 transcript copies. Won et al. [189] also developed an oligonucleotide-based microarray for the detection of foodborne viruses (including NoV, HAV, human rotavirus and human astrovirus), with the selected probes having a detection limit of 100 ng DNA for each virus. Even though microarray-based approaches have high throughput and are relatively inexpensive, problems such as high background (due to cross-hybridization) and limited detection range may hamper their application.

## 6. Omics-Based Approaches and Other Detection Methods

The omics-based approaches (including metagenomics, proteomics and metabolomics) have also been explored for NoV detection [190]. For instance, next generation sequencing has been applied for the subtyping of HuNoV with maximum resolution [191,192,193]. However, this technique cannot identify the infectivity of viruses, unless the viral genome is damaged. Mass spectrometry (MS) is another technique that has been applied for the identification of viral proteins and intact viruses [194]. The application of MS in the identification of NoV protein was first reported by Colquhoun et al. [195] using matrix-assisted laser desorption ionization (MALDI) coupled with a time-of-flight (TOF) and nano-electrospray ionization mass spectrometry (ESI-MS) in the detection of NoV VLPs in clinically relevant matrices. Even though the detection of intact proteins showed poor selectivity and sensitivity, peptide mass fingerprinting was able to detect up to 16 viral peptides and 0.1 pmol ~ 50 pmol of the 56 kDa NoV capsid protein in authentic standards of VLPs, in addition to more than 250 fmol of NoV capsid protein in stool extracts [195]. By comparison, Colquhoun [161] showed that MALDI-TOF MS performed more rapidly, was less expensive and could be used for the screening of relatively clean samples; while ESI-MS may be more suitable for complicated matrices and confirmatory analysis. Another interesting study conducted by Hellberg et al. [196] incorporated RT-PCR with ESI-MS for the detection and differentiation of NoV. This technique had a high sensitivity of 92% and specificity of 100% within one working day. Moreover, 98% of NoVs at the genogroup level and 75% of NoVs at the genotype level can be identified by this method, which could also be improved to obtain an even higher identification rate if more reference samples could be added to the database [196]. Direct ESI-MS has also been used for the quantitative study of the interaction between the P2 domain of GII.4 with HBGAs by Han et al. [25] Unfortunately, the high cost of the equipment and large space requirement limits the application of MS for the detection of NoV [194].

The omics-based methods are still under development and they have not been applied for NoV detection. The requirement of extraction of nucleic acids, proteins and metabolites from the NoV contained sample matrix may hinder the application of omics-based approaches in the rapid diagnosis and genotyping of NoV [190]. However, the omics-based approaches were described to have potentials for biomarker detection that could be applied for rapid kit development for NoV detection [190].

Aside from the aforementioned techniques, several other methods have also shown potential in NoV detection. For example, the capillary isoelectric focusing-whole column imaging detection (CIEF-WCID) method [197] has been used for determination of the isoelectric point (pI) values of NoV VLPs. This particular method introduces the possibility of differentiating different NoVs based on their pI values, as long as their pI values are different [197]. However, the CIEF-WCID method requires optimized sample preparation and extraction conditions to obtain high purity virus samples (without sample matrix or environmental contaminants), as well as high purity reagents to avoid impurity peaks. In addition, theoretical pI values of NoV VLPs based on the capsid sequence of VLPs were close, with a range of 5.2 to 5.7, though experimental determinations maybe slightly different [197]. Therefore, the application of CIEF-WCID method may have limitations for rapid detection and genotyping of NoV. Similarly, the dynamic light scattering (DLS) technique has also been investigated for NoV detection, however, high concentrations of purified capsids are required for the accurate determination of viruses by this technique [198]. The mechanism of using the DLS technique for NoV detection is that DLS can be used for detection of dispersed or aggregated viruses, as well as viral capsid proteins and dimers with the condition that the identity and purity of the analyte are known [198]. Virus aggregation is a sign of loss of capsid integrity due to the easy aggregation of disrupted capsids [198].

## 7. Future Perspective

Over the past 50 years, the methods for HuNoV detection have undergone dramatic improvements. Significant advances in the epidemiology, surveillance and diagnostics of HuNoVs have propagated its awareness and detection. However, challenges remain in currently available detection methods. First, the lack of broadly reactive ligands to be used for the detection of all HuNoV strains has hampered the development of a universal ligand-based method for HuNoV detection. Second, the limit of detection for HuNoV in these methods is still high. Further improvement is needed in the sensitivity for better application in real world situations. Third, several methods (i.e., PCR and ELISA) take hours in its detection of HuNoV, while others currently under development (e.g., biosensor methods) have yet to be applied to real world applications, though the detection could be achieved rapidly. Another major challenge is the ability to effectively concentrate a small number of viruses from large, complex food and environmental samples. Furthermore, methods still need to be developed for the real time detection of HuNoV, especially those that can differentiate infectious from non-infectious viruses and those capable of rapid subtyping.

Although currently no perfect real-time methods are available for detecting and subtyping infectious HuNoVs, the technology is evolving and has already introduced a number of new techniques into the field. Biosensor and microarray-based methods have especially shed light on the possible development of rapid and highly sensitive detection assays, as well as the differentiation of infectious and non-infectious viruses. However, more work is needed to improve sample preparation/removal of inhibitors, identification of strong but broadly reactive ligands, discrimination of infectious virus particles from noninfectious, and techniques for both rapidly detecting and subtyping virus portably. Over the course of half of a century an astounding amount of progress has been made in analytical and detection techniques for noroviruses, however challenges remain and more research is needed.

## Figures and Tables

**Table 1:** Comparison of different types of methods for human norovirus (HuNoV) detection.

Method	Cost	Time	Sensitivity	Specificity	Detection Limit	Advantages	Disadvantages
Electron microscopy (EM)	High	15 min ^1^	Low	Low	10^6^ viral particles/mL stool	Fast; capable of visually observation of viral morphology	Low sensitivity and specificity; laborious and expensive operation (including the requirement of trained personnel)
Enzyme-linked immunosorbent assay (ELISA)	Medium	60~90 min ^1^	31.6%~92.0%	65.3%~100.0%	10^4^~10^6^ viral particles/mL stool	Cheap reagent; long shelf-life; widely available	Measurement of enzyme activity may be complex and the enzyme activity may be affected by plasma constituents
Immunochromatography (ICG)	Medium	15 min	17.0%~83.0%	87.5%~100.0%	NA	Fast; long shelf life (12~24 months); ease of use; relatively low cost and relative ease in manufacturing	Varied performance
Real time RT-qPCR	High	40 min~3 h (with pre-extracted nucleic acid)	High	High	Around 10~100 gc/g sample	High sensitivity and specificity	Reagent-intensive; requirement of specific equipment
Biosensor	High	Short (Varied)	High	High	Varied	Potentials for point-of-care diagnostics	Sample preparation; matrix effects and system integration

^1^ According to Vinjé [11].

**Table 2:** Comparison of bio-recognition elements for HuNoV detection.

Bioreceptor	Availability	Cost ^1^	Component	Specificity	Sensitivity	Advantages	Disadvantages	Reference
Monoclonal antibody	Some are commercially available; some are currently available in research laboratories	Varied	large (~150 kDa) multimeric proteins	High	Medium	High specificity and selectivity	Produced from animal systems, more expensive; the binding conditions cannot be modulated; heat sensitive and binding irreversible; limited shelf-life	[16,17]
Aptamer	Available in some research laboratories; can be synthesized commercially upon request	$6–16/nmol ^2^	DNA oligonucleotide, single strand	High	Medium	Chemical synthesis; the binding conditions can be modulated; heat stable and recoverable; less expensive; long shelf-life	Rapid degradation of aptamers by nucleases in biological media or in blood; time- and labor-consuming; may also bind to molecules with a similar structure; require purified target molecules for generation	[18,19]
Porcine gastric mucin (PGM)	Easily available commercially	$3.84/g ^3^	Type A, H type 1 and Lewis ^b^ HBGA, other carbohydrates as well as protein (20%)	Low	High	Low cost, easily available, and broad reactivity	Low specificity, since it can also bind to other microbes; PGM also contains another broadly recognized receptor-sialic acid	[20,21,22]
Histo-blood group antigens (HBGAs)	Difficult to obtain since only one company produces HBGAs, and it′s possibly backordered	~ $260/mg ^4^	ABH, secretor and Lewis antigens	Low	High	Commercially available; bind to all NoVs except for a few genotypes	Low specificity, since they can also bind to other viruses and bacteria, including Rotavirus and rabbit hemorrhagic disease virus	[20]

^1^ Prices are in USD. ^2^ Price based on IDT listing for RNA synthesis. Available online: https://www.idtdna.com/pages/products/custom-dna-rna/dna-oligos/ultramer-dna-oligos (Accessed on 6 March 2020). ^3^ Price based on SigmaAldrich.com listing. Available online: https://www.sigmaaldrich.com/catalog/product/sigma/m1778?lang=en&region=US (Accessed on 10 Feburary 2020). ^4^ Price is based on H type 1 synthetic HBGA (Le^d^ (H type 1)-PAA-biotin) on Glycotech Corp. (Gaithersburg, MD). Available online: http://www.glycotech.com/probes/multivalbio.html (Accessed on 10 Feburary 2020). For other HBGAs, the price may be different.

**Table 3:** Biosensor methods for detection of norovirus and its surrogates.

Biosensor Method	Target	Platform	Detection Material	Ligand Chosen	Sample Type	Detection Time	Linear Range	Detection Limit	Specificity	Reference
SERS	FCV	Functionalized gold chips	AuNP	mAb	Cell culture	N/A	1.0 × 10^6^~2.5 × 10^8^ viruses/mL	1 × 10^6^ viruses/mL (or ~70 captured viruses)	N/A	[129]
SERS	MNV	Gold coated silicon wafers	Gold substrate	N/A	Mixture virus strains	N/A	N/A	Titer of 100	N/A	[130]
SERS- ICG	NoV VP1 protein	Polyethylene polyvinyl chloride base strip	Colloidal gold	Antibody	Centrifuged fecal specimen	~15 min	3~150 ng/mL detection range	0.5 ng/mL	Good	[131]
LSPR-based fluorescence	NoV VLPs	NP solution	AuNP and QDs	Antibody	N/A	~5 min	10~100 pg/mL	0.4 pg/mL	N/A	[132]
LSPR-induced optical sensor	NoV VLPs and HuNoV	Liquid	CdSeTeS QDs/AuNP nanocomposite	Antibody	NoV VLPs and clinically isolated NoV	Response time 1 min	10 fg/mL~1 ng/mL NoV VLPs and 100~100,000 copies/mL	12.1 fg/mL NoV VLPs and 95 copies/mL clinically isolated HuNoV	Superior	[133]
Plasmonic biosensor	NoV capsid and HuNoV	LSPR layer	AuNP	Affinity peptide	NoV capsid protein and HuNoV	N/A	10~10^5^ copies/mL	0.1 ng/mL NoV capsid protein in culture media and 9.9 copies/mL HuNoV	High	[134]
LSPR-amplified fluorescence assisted by magnetic field	NoV VLPs and HuNoV	Liquid	AuNP/MNP hybrid nanocomposites and CdSeS QDs	GII antibody	NoV VLPs in feces and feces containing HuNoV	N/A	1 pg/mL to 5 ng/mL NoV VLPs; 10^2^~10^7^ RNA copies/mL GII isolated clinical HuNoV	0.48 pg/mL NoV VLPs in feces; 84~934 copies/mL GII HuNoV	High	[135]
SPR	FCV	Au sensor chip	Thin gold layer	Antibody	Purified cell culture lysates or spiked oyster matrices	<15 min	3 × 10^4^~10^6^ TCID_50_ FCV/mL	10^4^ TCID_50_ FCV/mL	N/A	[136]
SPR	HuNoV (GII.4)	Au sensor chip	Polyacrylate beads	Con A	Spiked lettuce, strawberries, and milk	15 min	N/A	up to 10 RT-PCR units/mL	N/A	[137]
SPR-assisted fluorescence	NoV VLPs	Al film on polystyrene substrate	CdSe-ZnS-based quantum dot fluorescent dye	mAb and pAb	N/A	N/A	0.01~1 ng/mL	0.01 ng/mL (or 100 VLPs)	N/A	[138]
Fluorescence	NoV GII RNA	Liquid	AuNR@CdSeTe QDs	MB containing 20 bp complementary to NV RNA	Purified and mixed virus RNA	N/A	2~18 copies/mL	1.2 copies/mL	High	[139]
Fluorescence	Single NoV VLP (GII.4)	Lipid bilayer coated glass-bottom microtiter wells	Rhodamine-labeled lipid vesicle	H type 1 GSL	N/A	<2 h	12~200 fM	16 fM (single-molecule)	High	[140]
Fluorescence	FCV	Fabricated nanoporous membranes in glass microchannels	Protein A superparamagnetic beads and fluorescent liposomes	mAb and pAb	Purified and dialyzed virus	Within 2.5 h	N/A	1.6 × 10^5^ PFU/mL	N/A	[141]
Fluorescence	Single strand NoV RNA	96 well plates	Fluorescent F0F1-ATPase molecular motor containing ε-subunit antibody-streptomycin-biotin-probe	NoV RNA probe	Extracted RNA	Within 1 h	N/A	0.005 ng/mL	High	[142]
Fluorescence	NoV capsid protein	Paper-based microfluidic platform	MWCNT or GO	Aptamer	Spiked mussel samples	~10 min	13 ng/mL to 13 μg/mL	3.3~4.4 ng center dot per mL	High	[70]
Chemiluminescence	NoV GII capsid	Magnetic NP solution	GO/Fe_3_O_4_ nanocomposite	Modified aptamer	Tap water and artificial urine	30 min incubation time	0.16–10 μg/mL	80 ng/mL (in tap water)	High	[143]
BLI	NoV antibody	Octet BLI sensor	Co(III)-NTA	Avidin and His-tagged NVLPs	Human serum samples	10–15 min oxidation time	N/A	N/A	N/A	[144]
BLI	NoV antibody	Octet BLI sensor	Ni-NTA	NoV VLPs or NoV P-particles	Human serum samples	10-20 min with pre-functionalized sensors	N/A	Dilutions up to 1:100,000	N/A	[145]
BLI	NoV VLPs (GI.1 and GII.4)	Needle-shaped sensor	N/A	Antibodies	N/A	2 min	10~20 μg/mL	5 μg/mL	N/A	[146]
3D dual-view light sheet microscopy based	NoV GI capsid	Gold nanoarray on glass wafer	AuNS and AgNP	Antibody	NoV capsid spiked lettuce leaf	N/A	7.8 zM~240 aM	7.8 zM	Signal slightly increase towards other antigens	[147]
Photoluminescence based biosensor	NoV GII RNA	96-well plate	SiO_2_-coated CdZnSeS QD	Molecular beacon	Buffer and human serum	3 min hybridization time	2~16 copies/mL in buffer and 0~8 copies/mL in human serum	8.2 gc/mL in human serum and 9.3 gc/mL in buffer	high	[148]
Near-field illumination biosensor assisted by external magnetic field	NoV VLPs	Liquid cell	Magnetic bead and polystyrene bead	Antibody	Contaminated water	N/A	N/A	40 particles per 100 mu l in contaminated water	N/A	[149]
Nake-eye biosensor	HuNoV	Dot-blotting	Polyhedral Cu nanoshell deposited AuNPs	Antibody	Stool	Signal generation time 10 min	2.7 × 10^3^~2.7 × 10^5^ copies	2700 copies NoV in clinical stool samples	High	[150]
Silver-enhanced nanozyme-based colorimetric immunoassay	NoV VLP and GII.4 feces	Microtiter plate	Au/Ag NPs	GII antibody	Human feces	N/A	10~10^5^ pg/mL NoV VLP ; 10~10^4^ and 10^2^~10^5^ copies/mL fecal solution for NoV GII.3 and GII.4, respectively	10.8 pg/mL NoV VLP; 13.2~16.3 gc/mL fecal NoV GII.3 and GII.4 (or 132~163 gc/g feces)	High	[151]
Colorimetric NanoZyme aptasensor	MNV	Liquid	AuNP	AG3 Aptamer	Cell culture in presence of matrix	10 min	200−10,000 viruses/mL, or 1320−19,800 viruses/mL, or 3300−33,000 viruses/mL	200 viruses/mL (experimental) and 30 viruses/mL (calculated)	High	[69]
Electrochemical	GII NoV VLPs	PDMS microfluidic chip	GRP-AuNPs composite modified carbon electrode	Aptamer	Spiked blood samples and samples with other interferences	N/A	100 pM~3.5 nM	100 pM	High	[152]
Electrochemical	NoV RNA	Planar Pt-IDE	Au/iron-oxide MNP-decorated CNT	Probe RNA	N/A	N/A	1 pM~10 nM	8.8 pM	High	[153]
Electrochemical	NoV VLPs	Pt-IDE	Au/MNP-decorated GRPs	Antibody	N/A	N/A	0.1 pg/mL~1 ng/mL	1.16 pg/mL	High	[154]
Electrochemical	NoV capsid proteins or HuNoV	Three-electrode cell	Au electrode	Affinity peptide	Spiked fetal bovine serum	N/A	0.01~1000 μg/mL NoV capsid proteins; 1~10^3^ or 10^3^~10^6^ NoV	99.8 nM for NoV capsid proteins and 7.8 copies/mL for HuNoV	Varied	[155]
Electrochemical	NoV capsid	Chromium IDE fabricated glass substrate	Chromium IDE	N/A	Recombinant NoV capsid	5 min	N/A	2.5 ng/mL	N/A	[156]
Voltammetric electrochemical	NoV (GII.4)	PDMS bonded glass substrate	Au electrode	Con A and NoV antibodies	Lettuce	1 h	10^2^ and 10^6^ copies/mL	60 copies/mL	98%	[157]
Electrochemical	FCV	Fabricated nanoporous membranes in glass microchannels	Protein A superparamagnetic beads and electrochemical liposomes	mAb and pAb	Purified and dialyzed virus	2.5 h	N/A	3.2 × 10^6^ PFU/mL	N/A	[141]
Electrochemical	MNV	Fabricated silicon substrate	Au working electrode	Aptamer AG3	Cell culture	N/A	10~10^4^ PFU/mL	10 PFU/mL	N/A	[68]
Electrochemical	MNV	SPE	AuNP	Aptamer AG3	Cell culture	60 min	20~120 aM (ca. 360~2170 viral particles)	10 aM (~180 virus particles)	Not high	[67]
Impedance electrochemical	HuNoV GII.4	SPE	Au electrode	NoroBP-nonFoul (FlexL)_2_ peptide	Clinical HuNoV	Within 30 min	0 to 10^4^ copies/mL HuNoV GII.4; 10 to 10^5^ copies/mL extracted NoV from oysters	1.78 gc/mL HuNoV GII.4 and 2.47 gc/mL NoV from oysters	High	[158]
Field effect transistor based	NoV VLPs	Kapton films	Inkjet-printed graphene materials	Antibody	NoV VLPs	N/A	0.1 to 100 μg/mL NoV VLPs	~0.1 μg/mL	N/A	[159]
Photoelectrochemical	NoV RNA	An electrochemical workstation	CdSe–ZnO	DNA probe	Spiked diluted serum	N/A	0~5.10 nM NoV RNA	0.50 nM	N/A	[160]

Abbreviations: Surface-enhanced Raman spectroscopy (SERS); immunochromatography (ICG); localized surface-plasmon resonance (LSPR); surface plasmon resonance (SPR); feline calicivirus (FCV); murine norovirus (MNV); norovirus (NoV); recombinant norovirus-like particles (NoV VLPs); multi-walled carbon nanotubes (MWCNT); graphene oxide (GO); interdigitated electrode (IDE); Concanavalin A (Con A); polydimethylsiloxane (PDMS); glycosphingolipids (GSL); screen-printed electrode (SPE); biolayer interferometry (BLI); norovirus like particles (NoV VLPs); monoclonal antibody (mAb); polyclonal antibody (pAb); 50% tissue culture infective dose per mL (TCID_50_/mL); gold nanoparticle (AuNP); magnetic nanoparticle (MNP); quantum dots (QD); carbon nanotubes (CNT); aluminum (Al); gold (Au); graphenes (GRPs); three-dimensional (3D); gold nanospots (AuNSs); silver nanoparticles (AgNP); molecular beacon (MB).
